# Should I stay or should I go? The impact of working time and wages on retention in the health workforce

**DOI:** 10.1186/1478-4491-12-23

**Published:** 2014-04-23

**Authors:** Stephanie Steinmetz, Daniel H de Vries, Kea G Tijdens

**Affiliations:** 1 Department of Sociology & Anthropology, University of Amsterdam, Amsterdam, the Netherlands; 2 Amsterdam Institute for Advanced Labor Studies (AIAS), University of Amsterdam, Amsterdam, the Netherlands

**Keywords:** Commuting time, Health workforce retention, Intention to quit, Intention to stay, Job satisfaction, Remuneration, Survey data, Wage satisfaction, Working time

## Abstract

**Background:**

Turnover in the health workforce is a concern as it is costly and detrimental to organizational performance and quality of care. Most studies have focused on the influence of individual and organizational factors on an employee’s intention to quit. Inspired by the observation that providing care is based on the duration of practices, tasks and processes (issues of time) rather than exchange values (wages), this paper focuses on the influence of working-time characteristics and wages on an employee’s intention to stay.

**Methods:**

Using data from the *WageIndicator* web survey (*N* = 5,323), three logistic regression models were used to estimate health care employee’s intention to stay for Belgium, Germany and the Netherlands. The first model includes working-time characteristics controlling for a set of sociodemographic variables, job categories, promotion and organization-related characteristics. The second model tests the impact of wage-related characteristics. The third model includes both working-time- and wage-related aspects.

**Results:**

Model 1 reveals that working-time-related factors significantly affect intention to stay across all countries. In particular, working part-time hours, overtime and a long commuting time decrease the intention to stay with the same employer. The analysis also shows that job dissatisfaction is a strong predictor for the intention to leave, next to being a woman, being moderately or well educated, and being promoted in the current organization. In Model 2, wage-related characteristics demonstrate that employees with a low wage or low wage satisfaction are less likely to express an intention to stay. The effect of wage satisfaction is not surprising; it confirms that besides a high wage, wage satisfaction is essential. When considering all factors in Model 3, all effects remain significant, indicating that attention to working and commuting times can complement attention to wages and wage satisfaction to increase employees’ intention to stay. These findings hold for all three countries, for a variety of health occupations.

**Conclusions:**

When following a policy of wage increases, attention to the issues of working time—including overtime hours, working part-time, and commuting time—and wage satisfaction are suitable strategies in managing health workforce retention.

## Abstract (de)

**Abstract in German:**

**Hintergrund**

Hohe Personalfluktuation und Kündigungsraten sind im Gesundheitswesen aufgrund ihres negativen Einflusses auf die organisatorische Leistung sowie die Qualität der Pflege in zunehmendem Maße ein ernstes Problem. In diesem Zusammenhang haben Studien zur Personalfluktuation vor allem den Einfluss von individuellen und organisatorischen Faktoren untersucht. Da jedoch innerhalb des Gesundheitswesens Zeitkomponenten (z.B. Dauer der für verschiedene Aufgaben verfügbaren Zeit) eine noch wichtigere Rolle spielen als die Entlohnung jedes einzelnen Arbeitsschritts, konzentriert sich der vorliegende Artikel vor allem auf den Einfluss verschiedener Arbeitszeit- und Entlohnungsfaktoren auf die Absicht von Mitarbeitern, beim derzeitigen Arbeitgeber zu verbleiben.

**Methode**

Unter Verwendung gepoolter belgischer, deutscher und niederländischer Stichproben (2006-2012) der kontinuierlichen, weltweiten und mehrsprachigen *WageIndicator-*Onlineumfrage (N = 5323) untersucht die Studie anhand von drei logistischen Regressionsmodellen die Absicht von Mitarbeitern, bei ihrem derzeitigen Arbeitgeber im Gesundheitswesen zu verbleiben. Das erste Modell analysiert unter Kontrolle von soziodemographischen sowie berufs- und organisationsbezogenen Eigenschaften den Einfluss verschiedener Arbeitszeitfaktoren auf die Verbleibeabsicht. Unter Berücksichtigung der gleichen Kontrollvariablen testet das zweite Modell hingegen die Auswirkungen lohnbezogener Eigenschaften auf die Absicht zu bleiben, während das dritte Modell letztlich Arbeitszeit- und Lohnaspekte kombiniert.

**Ergebnisse**

Modell 1 zeigt, dass die arbeitszeitbezogenen Faktoren die Absicht, beim derzeitigen Arbeitgeber zu verbleiben, in allen drei Ländern signifikant beeinflussen. Insbesondere Teilzeitarbeit, Überstunden sowie eine lange Anfahrtszeit zum Arbeitsplatz verringern die Verbleibeabsicht. Die Analyse zeigt auch, dass neben Einflussfaktoren wie weibliches Geschlecht, mittlere bis hohe Bildung und kürzlich erfolgte Beförderung, vor allem Arbeitsunzufriedenheit ein starker Prädiktor für die Absicht ist, den Arbeitgeber zu verlassen. In Modell 2 zeigt sich, dass lohnbezogene Merkmale, wie z.B. ein niedriger Lohn oder höhere Lohnunzufriedenheit, die Verbleibewahrscheinlichkeit von Arbeitnehmern verringert. Der starke Lohn(un)zufriedenheitseffekt bestätigt dabei, dass nicht nur die Lohnhöhe sondern vor allem auch die subjektive Lohnzufriedenheit eine zentrale Rolle spielt. Unter Berücksichtigung aller Faktoren in Model 3 bleiben die oben genannten Effekte signifikant, was darauf hindeutet, dass Arbeitszeitfaktoren (u.a. auch Anfahrtszeiten) neben Lohnfaktoren einen wichtigen Beitrag zum Verständnis von Personalfluktuation im Gesundheitswesen leisten. Diese Ergebnisse gelten für alle drei untersuchten Länder und eine Vielzahl von Gesundheitsberufen.

**Schlussfolgerungen**

Die Analyse zeigt auf, dass in der politischen Diskussion neben Lohnerhöhungen vor allem auch Themen wie Arbeitszeit (einschließlich Überstunden, Teilzeit und Anfahrtszeit) sowie die subjektive Lohnzufriedenheit in den Vordergrund gerückt werden müssen. Diese Faktoren eröffnen alternative Strategien, um das Problem hoher Personalfluktuation im Gesundheitswesen anzupacken.

## Abstract (es)

**Abstract in Spanish:**

**Antecedentes**

La rotación de personal de salud es preocupante, ya que es costoso y perjudicial para el desempeño de la organización y la calidad de atención médica. La mayoría de los estudios se han centrado en los factores a nivel individual y de organización que influyen el renunciar el empleo. Inspirado por la observación de que la prestación de atención médica se basa en la duración de las prácticas, las tareas y procesos (cuestiones de tiempo) en lugar de los valores de cambio (salarios), este manuscrito se enfoca en la influencia de las características del tiempo de trabajo y los salarios en la intención de permanecer en el empleo.

**Métodos**

Utilizando datos de la encuesta por Internet del Indicador Salarial (N = 5,323), se estimaron tres modelos de regresión logística para determinar la intención del empleado de atención de la salud a permanecer en Bélgica, Alemania y Holanda. El primer modelo incluye características de tiempo de trabajo, mientras controla por un conjunto de variables sociodemográficas, categorías laborales, la promoción y diversas características relacionadas con la organización. El segundo modelo de prueba el impacto de las características relacionadas con los salarios. El tercer modelo incluye tanto el tiempo de trabajo como los aspectos relacionados con los salarios.

**Resultados**

Modelo 1 indica que los factores de trabajo relacionados con el tiempo afectan significativamente la intención de permanecer en el empleo a través de todos los países. Las horas de trabajo a tiempo parcial o tiempo extra y un tiempo largo de trayecto al trabajo disminuyen la intención de permanecer en el mismo empleo. El análisis también indica que la insatisfacción laboral es un fuerte predictor de la intención de renunciar el empleo, también el ser mujer, ser moderadamente o bien educada y el haber sido promovido dentro de la organización actual. En el Modelo 2, características relacionadas con los salarios demuestran que los empleados con un salario bajo o un bajo nivel de satisfacción sobre el salario son menos propensos a expresar la intención de quedarse. El efecto de la satisfacción salarial no es sorprendente; confirma que, además de un alto salario, la satisfacción salarial es importante. Al considerar todos los factores en el Modelo 3, todos los efectos siguen siendo significativos, que indica que el aumentar la intención de los empleados a quedarse requiere la atención al tiempo del trabajo y del trayecto al trabajo, además de la atención sobre los salarios y la satisfacción de salarios. Estas conclusiones son válidas para los tres países y a través de una variedad de profesiones de la salud.

Cuando se implementa una política de incrementar salarios para mejorar la satisfacción salarial, también se debe considerar otras estrategias para el manejo de la retención de personal de salud, como el de trabajar horas extras, trabajo a tiempo parcial y el tiempo del trayecto al trabajo.

## Background

Retention of people working in health care is a serious concern as turnover is enormously costly and detrimental to the organizational performance and the health system in general [1-3]. As indicated by the European Union’s 2012 Commission Action Plan for the EU Health Workforce, the health sector faces major challenges, owing to labour shortages, attrition and relatively low pay in some health occupations. While turnover rates differ across health cadres—for instance, nurses are less likely to leave the workforce than medical doctors and other specialized health professionals [4]—the replacement is costly because of the subsequent hiring and required training of new employees [3,5]. Moreover, high turnover rates have great implications not only for the quality, consistency and stability of services provided to people in need, but also for the working conditions of the remaining staff, e.g. increased workloads, disrupted team cohesion and decreased morale [6,7].

A variety of individual and organizational factors have been found to impact turnover. The main focus of this article, however, is on the question in how far aspects of working time and remuneration influence retention, or the intention to stay. The theoretical approach of this article is informed by the longstanding criticism of formal and bureaucratic organizations over the objectification, commodification and standardization of labour. In past decades, health care experts have theorized that these concepts have brought about a loss of humanism in medicine, depersonalization of care, and the replacement of holistic care with bureaucratic control [8]. Central to the theory is the notion that when a free worker sells his or her labour for an indeterminate time, he or she receives a money-wage or salary and forms a continuing relationship with an employer, which is formalized through institutional processes and structures. Marxist theorists have long argued that alienation occurs when in this process the labourer loses control over his or her labour and therefore becomes a commodity. In recent decades, the associated objectification has increasingly been equated with dehumanization because it involves a professional neutralization of agency of both patients and health workers. Timmermans and Almeling speak of “an erasure of authenticity, an alienation of identities, and a silencing or even displacement of the self and the social world” [8].

An application of wage-labour analysis to human resources for health needs to take into account that in the field of human services, labour value is based not only on a notion of abstract (clock) time indifferent to the type of activity and used as an exchange value [9]. Within the social services required, work is conducted much more from a processual (or concrete) time associated more with the use values of work, anchored in the duration of social practices, tasks and processes, rather than exchange values [9,10]. Paid work in health care is illustrative of this type of labour because, ideally, processes take as long as they take, and cannot easily be hurried, as care needs are unpredictable. In recent decades, however, the conditions of neoliberal globalization have tended to privilege labour as exchange value over labour as use value.

Previous studies focusing on turnover have neglected the impact of work-related aspects of working time and remuneration. Moreover, those studies examining the relation between wages and intention to quit are rather inconclusive, pointing towards a more complex relationship between wages and additional personal and organizational characteristics. However, as working time and wages are closely related to job satisfaction, as well as attrition (or migration) of employees within and across countries [11-14], there is a need to explore their interrelation in more detail to employ retention strategies effectively. In addition, while current studies on retention have been focused on individual health care occupations or single countries, less attention has been paid to whether the observed factors also apply for a greater variety of health occupations. To optimize retention strategies, it is important for organizations to understand whether the reasons for quitting or staying are the same for the different occupations or not. A similar reasoning can be applied in exploring cross-national differences of health care systems to better understand why some countries might be more attractive for health care workers than others. However, owing to a lack of comparative data [15], such cross-national comparisons are lacking.

### Factors influencing intention to quit or stay

In the framework of this article, we focus on ‘intention to stay’ rather than on actual attrition or turnover. This framing differs from the typical negatively framed questions asked in studies, which typically affirm leaving (e.g., “I am actively seeking other employment.”) rather than staying in one’s current position [16-20]. However, as this article focuses more on retention, it seems more logical to use an outcome that offers a long-term perspective on remaining with the same employer or not. Following the argument of Mor Barak *et al.*[21], focusing on ‘intention’ rather than actual behaviour seems reasonable for two reasons. First, before actually leaving the job, workers typically make a deliberate and conscious decision to do so [22]. In previous studies, intent to leave has been found to be a good proxy indicator for actual turnover [23-27]. Second, in a cross-sectional study, it is more practical to ask employees of their ‘intention to quit or stay’ than to actually track them down in a longitudinal study to see whether they have left or to conduct a retrospective study and risk hindsight biases [28].

As indicated above, the reasons that employees quit their jobs are manifold and have been examined since the 1950s. Subsequent studies have developed models based on theoretical approaches of different disciplines. As results are often rather inconclusive, depending on the theoretical approach, only a combination of different disciplinary perspectives (economic, sociological and psychological) can contribute to the understanding of the complex process leading to intention to quit [29,30]. In the context of this article, results based on groups of factors are briefly summarized, focusing on the health care sector.

Starting with *sociodemographic characteristics* of employees, only a few characteristics seem to meaningfully predict the intention to quit. In particular, age and education are significant predictors. Studies have shown that younger and better educated employees are more likely to leave their jobs to seek career advancement [6,31,32]. This particularly happens if there are limited career opportunities within the organization [28]. For the health workforce, the findings are inconclusive when differentiating by profession. While well-educated younger nurses are more in favour of developing their careers and older nurses are likely to be a more stable workforce [6,16,33,34], quitting behaviour was independent of educational level for other health occupations [33]. Moreover, it seems unclear whether the observed relation between age and intention to quit simply reflects age, rather than work experience and tenure [35]. A further consistent significant predictor for turnover in health care facilities is ethnicity, showing that white people have, possibly due to increased job mobility or opportunity, a higher turnover than persons who are members of minority groups [33]. With respect to gender or marital status, there is little evidence that these characteristics are linked to turnover [16,33,36-38], though having children at home correlates with turnover, especially for women [36,39]. This is confirmed for nurses, showing that kinship responsibilities involving home obligations, children, spouses and ageing parents affect the work and turnover habits of nurses, possibly requiring a change in work environment [6,40-42]. McKee *et al.*[39] find marital status to be indirectly related to intention to quit in that employees who are married are more satisfied with their jobs and feel more supported and less stressed than their unmarried colleagues.

Besides sociodemographic factors, many studies show that *professional perception*, in particular job satisfaction—defined as the extent to which one feels positively or negatively about one’s job [43]—is a rather consistent predictor of turnover behaviour [6,21,44-49]. Employees who are satisfied with their jobs are less likely to quit [18,50-54]. However, it has been questioned whether job satisfaction is a valid predictor of turnover [37,55-58], in particular, since it remains unclear whether the relationship is direct or indirect via the impact on professional and organizational commitment [46,59-61]. For example, several authors view turnover as a product of job satisfaction and commitment, which in turn are influenced by organizational factors, demographics and environmental factors, such as alternative job opportunities outside the organization [18,40,62-64]. Overall, it seems that the number of influencing variables that are dependent of the underlying theoretical models appear too complex to provide clarity.

Finally, intention to quit is also associated with *work-related characteristics,* such as organizational climate, including the quality of relationship among staff members [65,66] and perceptions of job insecurity, as they are closely linked to job satisfaction and performance [23,24,67,68]. In addition, research among nurses has shown that promotional opportunities, career development and lifelong learning activities promote job satisfaction and increased retention [6,69]. A similar positive effect has been found for organizational responsibilities and empowerment on intention to quit, as employees feel more valued by being given responsibilities [70,71].

While such factors seem to be associated with retention, research has shown that, by contrast, a consistently heavy workload increases job tension and decreases job satisfaction, which in turn increases the likelihood of turnover [6,32]. In this context, it has been demonstrated that, for the health workforce in particular, working time is a crucial variable. Studies have found that temporal burdens, such as overtime (e.g. long shifts) and irregular working times (weekends, nights and holidays) are related to anticipated turnover [39,72], while limitations on working hours and the provision of rest periods (more off-time, flexibility in shifts, more choice of shifts) have a direct positive impact, not only on the quality of services but also on the intention to quit [19,34,40].

While time plays a central role in the constitution of the employment relationship, wages are closely related, as they constitute a key exchange value within abstract, commodified labour time. Moreover, wages have long been assumed to be central to health service delivery, as they presumably affect job and life satisfaction, employment and working conditions, as well as attrition of employees. However, studies on the impact of wages on turnover in the health services context are inconclusive. A Taiwanese hospital study from Yin and Yang [40] finds that pay (salary, fringe benefits and night-shift benefits) is the strongest factor related to nurse turnover. In contrast, Hayes *et al.*[6] show in their literature review that the impact of wages appears to be mixed, and also depends on whether other types of financial benefit, such as bonuses, pensions, insurance, allowances, fellowships, loans and tuition reimbursement, are considered. Tai *et al.*[33] also report evidence that more affluent individuals might have less need or motivation to change jobs in order to improve their income status. While these studies focus on absolute wage levels, no studies were found that explore the impact of perceived satisfaction with wage and wage-related collective bargaining coverage on intentions to quit, whereas in most European Union Member States, wages are primarily moderated by collective bargaining.

### Research question and hypotheses

Against this background, the main objective of this article is to understand the relationship between working time and remuneration on intention to stay using cross-sectional survey data. In particular, the following research questions will be addressed:

1. What is the influence of working-time-related factors on intention to stay?

2. What is the influence of wages and wage-related factors on intention to stay?

First, it is assumed that full-time work will increase the chance of staying with an employer (H1) as the commitment of full-time workers to a job is assumed to be higher, possibly because labour is less explicitly measured by the hour. Furthermore, it is hypothesized that long and additional working hours (H2) as well as non-standard working hours (such as shifts and evening hours, H3) will decrease the intention to stay with an employer. In addition, it is assumed that long commuting times will decrease the intention to stay with an employer (H4).

With respect to wages, it is assumed that an increase in wages also increases the chances of staying with the employer (H5). In addition, as collective bargaining coverage is mostly perceived as a stable, thus attractive, working condition, it should also increase the likelihood of employees to stay with the employer (H6). Finally, it can also be expected that employees who are satisfied with their wage will have a higher chance of remaining with the employer, as the rewards offset the disadvantages of commodified labour time (H7).

## Methods

### Data

The data used in this study stem from the self-administered *WageIndicator* questionnaire, which is posted continuously at all national *WageIndicator* websites (http://www.wageindicator.org). The first *WageIndicator* website started in the Netherlands in 2001, and *WageIndicator* is operational today in 75 countries in five continents, receiving millions of visitors. The websites consist of job-related content, labour law and minimum wage information, VIP wages and a free salary check, presenting average wages for occupations based on the web survey data. Web traffic is high, owing to coalitions with media groups with a strong Internet presence, search engine optimization, web-marketing, publicity, mobile applications, and responding to visitors’ emails. The websites are consulted by employees, self-employed people, students, job seekers, individuals with a job on the side, and similarly for their annual performance talks, job mobility decisions, occupational choices or other reasons. In return for the free information provided, web visitors are invited to complete a voluntary questionnaire (two parts, each approximately ten minutes) with a lottery prize incentive. Between 1% and 5% of the visitors do complete the survey. Since the start of the survey, more than 1 million visitors to the website have provided valid information about their weekly, monthly or annual wages. The questionnaire is comparable across countries. It is in the national languages, adapted to country peculiarities, and asks questions about a wide range of subjects, including basic sociodemographic characteristics, wages and other work-related topics (see Additional file 1).

With respect to the quality of the data set, the voluntary nature of the survey is a challenge. In the scientific community, the increasing use of web surveys has triggered a heated debate on their quality and reliability for scientific use [73,74]. Arguments in favour of web surveys emphasize cost benefits, fast data collection, ease of processing results, flexibility of questionnaire design and the potential to reach respondents across national borders. The most obvious drawback is that they may not be representative of the population of interest. The sub-population with Internet access, the sub-population visiting the web survey’s website, and the sub-population deciding to complete the survey are quite specific, with respect to sociodemographic characteristics. In case of the *WageIndicator* data, several studies have shown that most web samples deviated to some extent from representative reference samples with regard to the common variables of age, gender and education [75-78]. It has also been demonstrated that the sample bias differs tremendously across countries, with higher selectivity in countries with lower Internet penetration rates and growth. To deal with the described problem, different adjustment techniques (e.g., poststratification weighting and propensity score adjustment) have been considered. To investigate the bias in the health care labour force, our sample could be compared with Eurostat’s labour force data for the years 2008 to 2012 (NLD until 2011) [79]. The comparison shows that, in all countries and in all years, the age group 20–49 was overrepresented in the web survey for both sexes, whereas the age group 50–59 was underrepresented. On average, overrepresentation for the age group 20–49 was 12% for the women and 11% for the men in Belgium, 6% for the women and 3% for the men in Germany, and 6% for the women and 7% for the men in the Netherlands. As the implementation of proportional weights does not change the outcome tremendously, we decide to use the unweighted data and consider the results as exploratory rather than representative.

The *WageIndicator* survey data provides detailed information on all relevant variables needed to explore retention. The analysis is limited to three countries, namely Belgium, the Netherlands and Germany. This choice is somewhat pragmatic, as these countries provided sufficient observations for the analysis, but is further justified by the fact that these are three north-western European neighbouring countries sharing cultural similarities, and all providing relatively high standardized wages (from $20/h to $26/h) across medical occupations [15]. A study of nurses commissioned by the European Commission in 2003 showed that the proportion of participants considering leaving nursing (several times a month or more) is, however, lower in the Dutch and Belgian samples (8.8% and 9.8%, respectively) than in the German sample (18.5%) [80]. Together, this selection of countries does bias this sample to the lower end of levels of intention to quit (12.4%), as compared with the European mean (15.6%). Only employed people, including apprentices, aged between 18 and 59 who work in a health-related occupation were included. We restricted age to people below 60 in order to filter out early retirement and people with possible health problems. Self-employed people are excluded because for these workers the intention to quit the job is most likely subject to other reasons than those given by employees. Cases who reported a gross hourly wage lower than €3.00 and above €400.00 are defined as outliers and therefore excluded. Being a continuous survey, the data from 2006 to 2012 could be pooled to obtain sufficient observations in the health-related occupations. All missing values as well as outliers were omitted from the analysis. The final total number, *N*, is 5,323 respondents with 797 respondents in Belgium to 2,621 respondents in the Netherlands.

### Operationalization and analytical strategy

As already indicated, the *dependent variable* is a dummy variable measuring the intention to stay, determined by asking whether a person expects to be working for the current employer in the next year (yes = 1; no or don’t know = 0). An alternative measure within the survey would have been whether a person had been actively seeking employment in the previous 4 weeks. However, as the focus of this paper is retention, it seemed more logical to use a variable that offered a rather long-term perspective on remaining with the same employer or not. A correlation analysis between the two variables revealed a moderate negative relationship, *r* = −0.57 (*p* ≤ 0.001, *N* = 5,323) indicating that those who reported that they would remain with the same employer were also not actively searching for a job. To cover *wages and wage-related aspects*, three measures were included: the logged gross hourly wage (minimum, €1.171; maximum €5.952), wage satisfaction (dissatisfied, neither nor (reference) and satisfied); and whether the organization was covered by a collective wage agreement (yes = 1, no or don’t know = 0). For *working-time characteristics*, four measures are considered: whether a person works full-time (1) or part-time (0) according to his or her self-assessment, whether a person works overtime, i.e. more than the usual hours agreed in the contract (yes = 1; no = 0), whether the person works irregular hours, such as shifts or evenings (yes = 1; no = 0) and how long a person has to commute one way to work (below 60 min = 0; above 60 min = 1).

To cover additional factors that are likely to have an impact on the intention to stay, the following variables were also included as controls: gender (women = 1; men = 0), education (low (reference), medium and high (for the classification of national educational categories into these three classes, see Additional file 2), age (18–59), migration status (native = 1, migrant = 0), having a partner (yes = 1, no = 0) and having one or more children (yes = 1, no = 0). As job satisfaction is a key predictor of the intention to stay and closely related to working time and wages, it is also included in the analysis as a categorical measure (dissatisfied; neither satisfied nor dissatisfied (reference); satisfied). To control for variations in the intention to stay across different health occupations, specific occupational dummy variables (medical doctors, nurses, pharmacists, technical pharmaceutical assistants, and others (reference)) were included. Further working conditions of respondents are also considered, such as having a permanent contract (yes = 1; no = 0), being in a supervisory position (yes = 1; no = 0), being an apprentice (yes = 1; no = 0), as well as organization size (below 100 = 0; 100 or over = 1) and working in the public sector (yes = 1; no = 0). To explore the impact of alternative employment opportunities on the likelihood of staying, the absolute growth unemployment rates between 2006 and 2012 were included for each of the three countries, resulting in a quasicontinuous variable (minimum −1.6; maximum, 1.1). This allowed us to control for country- and time-specific variations (see Table 1).

**Table 1:** Descriptive statistics for the complete sample

**Variable**	**Mean**	**SD**	**Minimum**	**Maximum**
Expect to be with the same employer	0.591	0.492	0	1
Full-time	0.523	0.499	0	1
Non-standard hours	0.662	0.473	0	1
Overtime	0.388	0.487	0	1
Commuting time	0.042	0.200	0	1
Gross hourly wage (log)	2.808	0.468	1.1	5.9
Wage dissatisfaction	0.400	0.490	0	1
Neither satisfied by wage nor dissatisfied	0.307	0.461	0	0
Wage satisfaction	0.293	0.455	0	1
Covered by collective agreement	0.714	0.452	0	1
Job dissatisfaction	0.152	0.359	0	1
Neither satisfied by job nor dissatisfied	0.225	0.418	0	1
Job satisfaction	0.623	0.485	0	1
Women	0.798	0.402	0	1
Age	38.582	10.723	18	59
Low education	0.220	0.415	0	1
Medium education	0.502	0.500	0	1
High education	0.278	0.448	0	1
Having a partner	0.655	0.476	0	1
Having one or more children	0.584	0.493	0	1
Native	0.938	0.242	0	1
Medical doctor	0.040	0.196	0	1
Nurse	0.310	0.463	0	1
Pharmaceutical occupation	0.034	0.181	0	1
Other health occupation	0.616	0.487	0	1
Permanent contract	0.821	0.383	0	1
Promotion in current job	0.802	0.398	0	1
Supervisor position	0.196	0.397	0	1
Apprentice	0.017	0.130	0	1
Organization size	0.510	2.702	0	1
Public employment	0.200	0.400	0	1
Unemployment growth in country	−0.284	0.662	−1.2	1.1

Source: *WageIndicator* data for Belgium, Germany and the Netherlands, 2006–2012 (unweighted), *N* = 5,323.

As can be seen from Table 1, around 60% of all respondents expected to be with the same employer next year. The mean age in the sample was approximately 39 years, and the group were dominated by women (80%) and natives (94%). Most of the respondents worked either as nurses (31%) or in ‘other health occupations’ (62%). With respect to the main explanation variables, it can be seen that only 52% of the respondents had a full-time contract, 66% worked non-standard hours, and 39% worked overtime. Interestingly, while 40% of the sample reported wage dissatisfaction, 62% indicated satisfaction with the job.

To test the hypotheses, three binary multivariate logistic regression models were estimated (M1–M3). The enumerated variables were introduced, starting with Model 1 to test whether the expected relation between working time and intention to stay could be observed. In Model 2, the effect of wages and wage-related measures on intention to stay were tested. Model 3 included all relevant variables, to test the relationship between working time and wages.

## Results

### Bivariate findings

**Figure 1:**
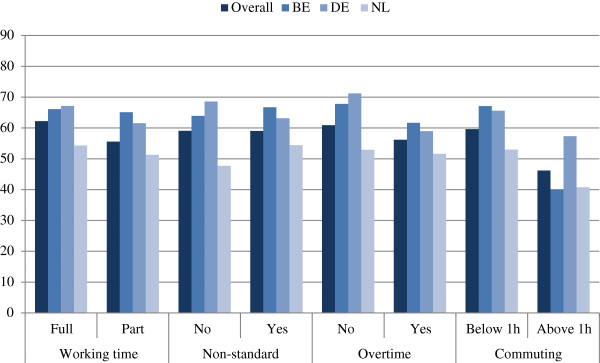
**Percentage of respondents intending to remain with the same employer within the next year in relation to working-time-related factors by country.** Source: *WageIndicator* data for Belgium, Germany and the Netherlands, 2006–2012 (unweighted), *N* = 5,323.

**Figure 2:**
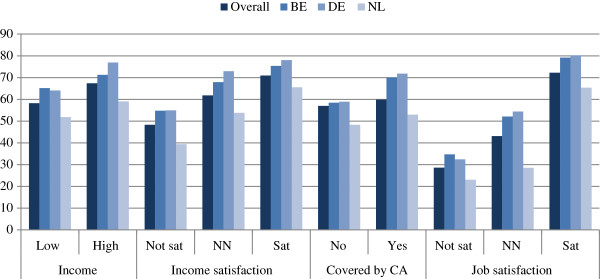
**Percentage of respondents intending to remain with the same employer within the next year in relation to wage-related factors by country.** Source: *WageIndicator* data for Belgium, Germany and the Netherlands, 2006–2012 (unweighted), *N* = 5,323.

Figures 1 and 2 show the percentage of respondents indicating that they would remain with the same employer in the next year in relation to working time and remuneration across the three countries. In general, the figures reveal that the intention to stay was lowest in the Netherlands for all considered variables with the exception of a one-way commuting time above 60 min, for which Belgium had a 2 percentage point lower rate.

When looking more closely at the pattern for the intention to stay with respect to working-time characteristics, Figure 1 shows that, in the Netherlands, it seems to be lowest for people with a commuting time above an hour, followed by people working non-standard working hours, part-time or overtime hours. For Germany, the intention to stay is higher overall but the pattern is comparable to the Netherlands, with the exception that there is a higher share of people with non-standard working hours who seem to intend to stay with the same employer. Finally, Belgium is somewhat in between the Netherlands and Germany but its pattern follows that of Germany more closely.

Continuing with the relation between intention to stay and wages, as well as wage-related factors, Figure 2 clearly shows that across all countries the intention to stay is lowest among those who are dissatisfied with their wage followed by people where the organization for which they are working is not covered by a collective agreement. As expected, the percentage for staying with the same employer is higher for respondents with a high wage, a high wage satisfaction and where the organization is covered by a collective agreement. With respect to country differences, as indicated previously, the Netherlands again stands out for all wage-related variables. However, the described pattern is the same across countries. Moreover, Figure 2 also shows that the share of respondents who intend to stay with the same employer within the next year is lowest for those with the lowest job satisfaction. This confirms previous findings, showing the importance of job satisfaction to retention.

### Multivariate findings

The results of the multivariate logistic regression analyses are presented in Table 2. Starting with the effect of working-time-related variables it becomes evident from Model 1 (M1) that even after controlling for sociodemographic and work-related variables, as well as job satisfaction, working-time characteristics are important for intention to stay (to remain with the same employer within the next year). In particular, working overtime (more hours than agreed in the contract) and a long commuting time significantly reduce the log-odds of a person remaining with the same employer. Conversely, the strong positive effect of full-time employment indicates that employees with a full-time job have a higher intention of remaining with the same employer in comparison to employees with a part-time job. With respect to non-standard working hours, no significant association could be observed at the 5% significance level.

**Table 2:** Log-odds on the probability of having the same job next year (intention to stay)

	**(M1)**	**(M2)**	**(M3)**
Full-time (yes =1)	0.334^***^ (0.069)		0.341^***^ (0.069)
Non-standard hours (yes =1)	0.063 (0.072)		0.074 (0.072)
Overtime (yes =1)	−0.190^**^ (0.065)		−0.156^*^ (0.065)
Commuting time (one way above 1 h =1)	−0.553^***^ (0.155)		−0.571^***^ (0.156)
Gross hourly wage (log)		0.158^*^ (0.076)	0.169^*^ (0.076)
Wage dissatisfaction (Reference: neither satisfied nor dissatisfied)		−0.248^***^ (0.075)	−0.247^**^ (0.076)
Wage satisfaction		0.210^*^(0.083)	0.200^*^ (0.083)
Covered by collective agreement (yes =1)		−0.124 (0.077)	−0.096 (0.078)
Constant	−0.876 (0.522)	−1.055^*^ (0.509)	−1.295^*^ (0.537)
Bayesian Information Criterion	−39294.3	−39289.0	−39298.8
Log likelihood	−3080.8	−3083.4	−3061.3

Standard errors in brackets, *P < 0.05, **P < 0.01, ***P < 0.00; in all models, we control for sociodemographic and work-related characteristics as well as for the absolute unemployment rate (see Additional file 3). Source: *WageIndicator* data for Belgium, Germany and the Netherlands, 2006–2012 (unweighted), *N* = 5,323.

Turning to Model 2 (M2), the consideration of the wage and wage-related factors is also relevant in explaining the intention to stay. As assumed, an increase in the gross hourly wage as well as a high level of wage satisfaction in comparison with a neutral level significantly increases the log-odds of staying with the same employer, while a higher wage dissatisfaction level significantly decreases the intention of people to stay with their employer within the next year in comparison with people with a neutral level of job satisfaction. With respect to the effect of collective agreement coverage, no significant association could be observed at the 5% significance level.

In addition, both models M1 and M2 reveal that, in line with previous studies, job dissatisfaction in comparison with a neutral level of job satisfaction is a strong predictor of the intention to leave an employer, while higher job satisfaction in comparison with a neutral level of job satisfaction increases retention. Moreover, the findings show that being a woman, or being moderately or highly educated in comparison with poorly educated, as well as being promoted in the current organization, also significantly reduces the intention to stay. This might be because moderately and highly educated people, as well as promoted people (where their good job performance has been confirmed by means of a promotion), might perceive more job opportunities and hence believe that they might find a better job within a year’s time. On the other hand, having a partner, a permanent contract or a public sector employment increases the intention to stay with the employer (see Additional file 3).

In the final Model 3 (M3), all factors are included in the analysis to test whether the effects previously observed remain significant. While most of the working-time- and wage-related effects slightly decreased or increased, they all remained significant. As a result, it can be concluded that the present analysis supports H1, H2 and H4, but not H3.

Turning to the wage-related factors, M3 reveals that the current analysis supports H5 and H7. Higher levels of wage satisfaction, as well as an increase in the gross hourly wage, significantly increase intention to stay. Conversely, the effect for collective agreement remains non-significant, indicating that wage setting through collective bargaining—at least in this analysis—does not affect the intention to stay or to quit. Hence, H6 is not supported.

Finally, when reflecting on the impact of the discussed explanation variables in relation to other relevant variables considered in the model (see Additional file 3), the greatest effects on the intention to leave the employer can be observed for people with a higher job dissatisfaction (in comparison with a neutral level), followed by people who have to commute longer than an hour one way and by better educated people. By contrast, the most important factors for the intention to stay seem to be a high level of job satisfaction, followed by a permanent contract and having a partner.

## Discussion and conclusions

In the framework of this article it has been argued that, besides job satisfaction, other work- and sociodemographic-related variables—in particular, working-time-related measures and wages—have to be taken into account when analysing retention in the health workforce. The main objective has been to gain a better understanding of the relationship between working time and remuneration on the intention to stay with the current employer within the coming year using survey data of health care employees for three West European countries. In this context, two research questions have been formulated:

1. *What is the influence of working-time-related factors on the intention to stay?*

In this respect, the analysis has revealed that working-time-related factors affect intention to stay across all countries. In particular, working part-time hours or overtime, as well as a long commuting time, decreases the intention to stay. While the effect of ‘overtime’ confirms previous results for nurses and doctors, the study shows that it also seems important to consider commuting time. While organizations can only marginally influence the location where people want to live, remuneration or other compensation schemes, such as adjustments in working time for those with long commuting times, might have to be further discussed among personnel departments. Conversely, this study could not confirm that non-standard working hours decreases the intention to stay. While studies with nurses have shown that non-standard working hours increase the intention to quit, the recent findings might be explained by the consideration of a broader variety of health occupations, in which such factors are less important.

2. *What is the influence of wages and wage-related factors on the intention to stay?*

As already indicated, prior studies on the impact of wages have been rather inconclusive. In the context of this study, in particular, the aspect of wage satisfaction and collective agreement coverage have been examined. The findings show that, in particular, employees with a higher wage or a high wage satisfaction are more likely to express an intention to stay. The effect of wage satisfaction—thus far rarely taken into account—is not surprising, but also shows that besides a high wage, satisfaction with a wage is essential when analysing retention in the health workforce.

Overall, these findings confirm the significance of the relationship between working-time-and wage-related factors (besides the well-known factors of, for instance job satisfaction) in efforts to increase intention to stay in the health services sector. In light of the critique of Colley *et al*. [9] that in late-capitalism the commodification of time restricts learning and promotes wages (exchange values) over caring for people (use values), these data show a need for further research on ‘temporality’ in human resources for health. In this context, it appears to be advisable that health service managers and policy makers pay more attention to the importance of employees’ working hours and working time, including, in particular, commuting time as well as the way in which working time interacts with personal wage satisfaction. In addition, trade unions may place more emphasis on perceived wage satisfaction in collective bargaining or permanent contracts [81]. Furthermore, and what is beyond the scope of this study, further analysis should explore the relation between working time and wages, wages and wage satisfaction as well as wage satisfaction and job satisfaction.

Finally, certain limitations of the study must be mentioned. Like much of the existing literature in human resources for health, this analysis is based on cross-sectional rather than longitudinal data. As a result, we were not able to measure actual turnover, although there is significant empirical evidence linking intention to quit with actual leaving in other settings. Cross-sectional studies may also be biased, because they only capture the views of health workers who are currently in service (and not those who have quit). More longitudinal research is an important priority to address these limitations. Moreover, even though the WageIndicator data offer a richness on wage and working-time-related variables associated with intention to stay (such as various bonuses and subjective stress factors), the large quantity of missing data on these variables rendered it impossible to include them in the analysis. Future studies, however, should extend these models with even more detailed information on wages and working time. In addition, as the analysis is based on a voluntary survey, the findings should be considered exploratory, although contributing to the understanding of retention in the health workforce.

## Competing interests

The authors declare no competing interests.

## Authors’ contributions

SS conducted the analyses of the survey data, DdV contributed to the literature review, and KT contributed to the formulation of hypotheses. All authors supported the design of the paper, reviewed and approved the final manuscript.

## Authors’ information

Stephanie Steinmetz is an assistant professor at the Department of Sociology and Anthropology at the University of Amsterdam and an affiliated senior researcher at the Erasmus Studio Rotterdam and AIAS. Her main research interests are (web) survey methodology, gender inequalities, comparative labour market research and quantitative research methods.

Daniel H. de Vries is an assistant professor at the Department of Sociology and Anthropology at the University of Amsterdam, and affiliated with the Center for Social Science and Global Health and AIAS. He was previously Research and Evaluation Manager at USAID’s Capacity Project, a human resources for health strengthening project.

Kea G. Tijdens is a research coordinator at Amsterdam Institute of Advanced Labor Studies (AIAS) at the University of Amsterdam, and a Professor of Women and Work at the Department of Sociology, Erasmus University Rotterdam. She is the scientific coordinator of the continuous *WageIndicator* web survey on work and wages. Her research interests are wage setting processes, working time and occupations.

## Supplementary Material

Additional file 1 **Stylized questionnaire.** The codebook is available for download; for academic research the data are available for free from the Forschungsinstitut zur Zukunft der Arbeit (IZA), Bonn, Germany, http://idsc.iza.org/?page=27&id=59. Click here for file

Additional file 2 Educational mapping: Belgium, the Netherlands, Germany. Click here for file

Additional file 3 Complementary tables. Click here for file
