# Supplementary material for: Should I stay or should I go? The impact of working time and wages on retention in the health workforce
Source: Hum Resour Health. 2014 Apr 23;12:23. doi: 10.1186/1478-4491-12-23 (PMC4021570; doi:10.1186/1478-4491-12-23)
Supplement: Additional file 3 — Complementary tables. [file 1478-4491-12-23-S3.docx]

| **Table 1. Description and recoding of variables**   \| **Variable** \| **Description** \| \| --- \| --- \| \| Intention to stay \| Responses to question whether respondent expects to be with the same employer next year. (Yes=1, No+don’t know=ref). \| \| *Wage and wage-related variables* \| \| \| Gross hourly wage (log) \| The gross hourly wages are computed from the reported weekly working hours, the reported gross wages and the reported pay period. For the cases with information about net hourly wages only, the gross hourly wages are computed based on the annual country average between gross and net wages. Then the hourly wages are converted into a standardized hourly wage in US dollars using purchasing power parities on an annual basis. \| \| Wage satisfaction \| Categorical dummified variable distinguishing between respondents who are highly dissatisfied and dissatisfied with their wage (1), satisfied and highly satisfied with their wage (2) and neither nor (ref). \| \| Collective agreement coverage \| Dummy-coded variable distinguishing whether the firm is covered by a collective agreement (=1.) or not (=ref.) \| \| *Working time variables* \|  \| \| Working time \| Dummy-coded variable where respondents self-define whether they work full-time(=1) or part-time (ref.) \| \| Non-standard hours \| Dummy-coded variable distinguishing between non-standard working hours such as shifts and evenings (=1) and standard working hours (=ref.) \| \| Overtime \| Dummy-coded variable distinguishing between respondents working normally more than agreed in contract (=1) and not (ref.) \| \| Commuting time \| Dummy-coded variable distinguishing between respondents commuting one way each day < 60 min (ref.) and those commuting one way each day 60 min or more (=1). \| \| *Controls* \|  \| \| Woman \| Gender, recoded (female=1, male=ref.) \| \| Age/ Age^2^ \| Continuous variable for age ranging from 18-59 \| \| Education level \| Categorical dummified variable distinguishing between low (ref.), medium and high education based on the ISCED classification. For each country the detailed national classification has been mapped to the ISCED classification. For details see the Additional File 3_ Educational mapping. \| \| Child \| Dummy-coded variable distinguishing between respondents having a child (=1) and no child (=ref). \| \| Native \| Dummy-coded variable distinguishing between people born in the country of survey (native=1) or not (foreign=ref) \| \| Job satisfaction \| Categorical dummified variable distinguishing between respondents who are highly dissatisfied and dissatisfied with their job (1), satisfied and highly satisfied with their job (2) and neither nor (ref). \| \| Type of health occupations \| Categorical dummified variable distinguishing between Medical doctors (1), Nurses (2), Pharmacists (3) and ‘other health occupations (ref). In the reference category occupations like, physiotherapists, medical and pharmaceutical technicians, personal care workers, health care administration and managers etc. \| \| Type of contract \| Dummy-coded variable distinguishing between a permanent (=1) and a temporary (ref.) contract \| \| Supervisor position \| Dummy-coded variable distinguishing between having a supervisory position (=1) or not (ref.) \| \| Apprentice \| Dummy-coded variable distinguishing between having an apprentice (=1) or not (ref.) \| \| Promoted in firm \| Dummy-coded variable distinguishing between whether the respondent has been promoted in the current firm (yes=1, no=ref.) \| \| **Variable** \| **Description** \| \|  \|  \| \| Organization size \| Dummy-coded variable distinguishing between organizations with < 100 employees (=ref.) and organizations with >=100 employees (=1). \| \| Public sector \| Dummy-coded variable distinguishing between public (=1) and private (=ref.) sector employment \| \| Unemployment growth \| Continuous variables representing the unemployment percentages per year and per country, based on Eurostat data, ‘Unemployment rates by sex, age and nationality (%)’, assessed 21-AUG-2013. \| |
| --- | --- | --- | --- | --- | --- | --- | --- | --- | --- | --- | --- | --- | --- | --- | --- | --- | --- | --- | --- | --- | --- | --- | --- | --- | --- | --- | --- | --- | --- | --- | --- | --- | --- | --- | --- | --- | --- | --- | --- | --- | --- | --- | --- | --- | --- | --- | --- | --- | --- | --- | --- | --- | --- | --- | --- | --- |

**Table 2: Descriptive statistics, overall and for each country**

|  | **Overall** | | **NL** | | **DE** | | **BE** | |  |  |
| --- | --- | --- | --- | --- | --- | --- | --- | --- | --- | --- |
| **Variable** | **Mean** | **SD** | **Mean** | **SD** | **Mean** | **SD** | **Mean** | **SD** | **Min** | **Max** |
| Expect to be with the  same employer | 0.591 | 0.492 | 0,525 | 0,499 | 0,653 | 0,476 | 0,657 | 0,475 | 0 | 1 |
| Full-time | 0.523 | 0.499 | 0,395 | 0,489 | 0,667 | 0,472 | 0,619 | 0,486 | 0 | 1 |
| Non-standard hours | 0.662 | 0.473 | 0,712 | 0,453 | 0,599 | 0,490 | 0,649 | 0,478 | 0 | 1 |
| Overtime | 0.388 | 0.487 | 0,336 | 0,472 | 0,482 | 0,500 | 0,050 | 0,218 | 0 | 1 |
| Commuting time | 0.042 | 0.200 | 0,041 | 0,199 | 0,039 | 0,195 | 0,050 | 0,218 | 0 | 1 |
| Gross hourly wage (log) | 2.808 | 0.468 | 2,845 | 0,442 | 2,770 | 0,498 | 2,777 | 0,467 | 1.1 | 5.9 |
| Wage dissatisfaction | 0.400 | 0.490 | 0,347 | 0,476 | 0,492 | 0,500 | 0,355 | 0,479 | 0 | 1 |
| Neither nor | 0.307 | 0.461 | 0,338 | 0,473 | 0,264 | 0,441 | 0,309 | 0,462 | 0 | 0 |
| Wage satisfaction | 0.293 | 0.455 | 0,316 | 0,465 | 0,244 | 0,429 | 0,336 | 0,473 | 0 | 1 |
| Covered by collective agreement | 0.714 | 0.452 | 0,898 | 0,303 | 0,497 | 0,500 | 0,629 | 0,483 | 0 | 1 |
| Job dissatisfaction | 0.152 | 0.359 | 0,136 | 0,343 | 0,172 | 0,377 | 0,156 | 0,363 | 0 | 1 |
| Neither nor | 0.225 | 0.418 | 0,194 | 0,395 | 0,261 | 0,440 | 0,241 | 0,428 | 0 | 1 |
| Job satisfaction | 0.623 | 0.485 | 0,670 | 0,470 | 0,567 | 0,496 | 0,604 | 0,489 | 0 | 1 |
| Women | 0.798 | 0.402 | 0,869 | 0,338 | 0,694 | 0,461 | 0,813 | 0,390 | 0 | 1 |
| Age | 38.582 | 10.723 | 37,572 | 11,169 | 41,078 | 10,033 | 35,939 | 9,597 | 18 | 59 |
| Low education | 0.220 | 0.415 | 0,152 | 0,359 | 0,387 | 0,487 | 0,046 | 0,211 | 0 | 1 |
| Medium education | 0.502 | 0.500 | 0,569 | 0,495 | 0,513 | 0,500 | 0,253 | 0,435 | 0 | 1 |
| High education | 0.278 | 0.448 | 0,279 | 0,448 | 0,100 | 0,300 | 0,700 | 0,458 | 0 | 1 |
| Having partner | 0.655 | 0.476 | 0,634 | 0,482 | 0,649 | 0,477 | 0,734 | 0,442 | 0 | 1 |
| Having child | 0.584 | 0.493 | 0,563 | 0,496 | 0,607 | 0,489 | 0,597 | 0,491 | 0 | 1 |
| Native | 0.938 | 0.242 | 0,935 | 0,247 | 0,943 | 0,231 | 0,935 | 0,247 | 0 | 1 |
| Medical doctor | 0.040 | 0.196 | 0,011 | 0,105 | 0,091 | 0,287 | 0,014 | 0,117 | 0 | 1 |
| Nurse | 0.310 | 0.463 | 0,309 | 0,462 | 0,318 | 0,466 | 0,297 | 0,457 | 0 | 1 |
| Pharmaceutical | 0.034 | 0.181 | 0,028 | 0,166 | 0,028 | 0,165 | 0,066 | 0,249 | 0 | 1 |
| Other health occupation | 0.616 | 0.487 | 0,652 | 0,477 | 0,564 | 0,496 | 0,622 | 0,485 | 0 | 1 |
| Permanent contract | 0.821 | 0.383 | 0,808 | 0,394 | 0,806 | 0,395 | 0,901 | 0,299 | 0 | 1 |
| Promotion in current job | 0.802 | 0.398 | 0,823 | 0,382 | 0,824 | 0,381 | 0,681 | 0,466 | 0 | 1 |
| Supervisor position | 0.196 | 0.397 | 0,163 | 0,369 | 0,256 | 0,437 | 0,163 | 0,370 | 0 | 1 |
| Apprentice | 0.017 | 0.130 | 0,026 | 0,159 | 0,012 | 0,109 | 0 | 0 | 0 | 1 |
| Organization size | 0.510 | 2.702 | 0,556 | 0,497 | 0,476 | 0,500 | 0,445 | 0,497 | 0 | 1 |
| Public employment | 0.200 | 0.400 | 0,200 | 0,400 | 0,224 | 0,417 | 0,146 | 0,353 | 0 | 1 |
| Unemployment growth | -0.284 | 0.662 | -0,120 | 0,756 | -0,499 | 0,484 | -0,310 | 0,536 | -1.2 | 1.1 |

Source: WageIndicator data for Belgium, Germany and the Netherlands, 2006 – 2012 (unweighted), N=5,323

**Table 3. Log-odds on the probability of having the same job next year (intention to stay)**

|  | (M1) | (M2) | (M3) |
| --- | --- | --- | --- |
| Full-time (yes =1) | 0.334^***^ |  | 0.341^***^ |
|  | [0.069] |  | [0.069] |
| Non-standard hours (yes =1) | 0.063 |  | 0.074 |
|  | [0.072] |  | [0.072] |
| Overtime (yes =1) | -0.190^**^ |  | -0.156^*^ |
|  | [0.065] |  | [0.065] |
| Commuting time (one way above 1h =1) | -0.553^***^ |  | -0.571^***^ |
|  | [0.155] |  | [0.156] |
| Gross hourly wage (log) |  | 0.158^*^ | 0.169^*^ |
|  |  | [0.076] | [0.076] |
| Wage dissatisfaction |  | -0.248^***^ | -0.247^**^ |
| *Ref. Neither nor* |  | [0.075] | [0.076] |
| Wage satisfaction |  | 0.210^*^ | 0.200^*^ |
|  |  | [0.083] | [0.083] |
| Cov. by collective agreement (yes =1) |  | -0.124 | -0.096 |
|  |  | [0.077] | [0.078] |
| Job dissatisfaction | -0.647^***^ | -0.611^***^ | -0.622^***^ |
| *Ref. Neither nor* | [0.101] | [0.101] | [0.102] |
| Job satisfaction | 1.349^***^ | 1.243^***^ | 1.245^***^ |
|  | [0.074] | [0.076] | [0.076] |
| Women(yes =1) | -0.314^***^ | -0.387^***^ | -0.295^***^ |
|  | [0.085] | [0.082] | [0.085] |
| Age | -0.007 | -0.015 | -0.008 |
|  | [0.025] | [0.025] | [0.026] |
| Age^2^ | 0.0003 | 0.0003 | 0.0003 |
|  | [0.0003] | [0.0003] | [0.0003] |
| Medium education | -0.213^**^ | -0.241^**^ | -0.237^**^ |
| *Ref. Low education* | [0.082] | [0.083] | [0.0843] |
| High education | -0.280^**^ | -0.346^***^ | -0.339^***^ |
|  | [0.093] | [0.096] | [0.096] |
| Partner (yes =1) | 0.432^***^ | 0.378^***^ | 0.405^***^ |
|  | [0.069] | [0.069] | [0.069] |
| Children (yes =1) | 0.052 | 0.024 | 0.069 |
|  | [0.082] | [0.081] | [0.082] |
| Native (yes =1) | 0.017 | 0.022 | 0.005 |
|  | [0.130] | [0.129] | [0.130] |
| *Ref. Other health occup.* |  |  |  |
| Medical doctor | 0.372^*^ | 0.321 | 0.303 |
|  | [0.172] | [0.173] | [0.174] |
| Nurse | -0.057 | -0.030 | -0.062 |
|  | [0.073] | [0.069] | [0.073] |
| Pharmacists | -0.198 | -0.153 | -0.197 |
|  | [0.172] | [0.172] | [0.173] |

|  | (M1) | (M2) | (M3) |
| --- | --- | --- | --- |
| Permanent contract (yes =1) | 0.708^***^ | 0.732^***^ | 0.702^***^ |
|  | [0.083] | [0.083] | [0.084] |
| Supervisory position (yes =1) | 0.141 | 0.137 | 0.098 |
|  | [0.084] | [0.083] | [0.085] |
| Apprentice (yes =1) | 0.022 | 0.122 | 0.094 |
|  | [0.244] | [0.243] | [0.246] |
| Promoted in firm (yes =1) | -0.305^***^ | -0.320^***^ | -0.306^**^ |
|  | [0.083] | [0.082] | [0.083] |
| Organization size (above 100 =1) | 0.089 | 0.093 | 0.075 |
|  | [0.065] | [0.068] | [0.068] |
| Public (yes =1) | 0.260^**^ | 0.258^**^ | 0.254^**^ |
|  | [0.080] | [0.081] | [0.082] |
| Unemployment growth | 0.065 | 0.068 | 0.068 |
|  | [0.049] | [0.048] | [0.049] |
| Constant | -0.876 | -1.055^*^ | -1.295^*^ |
|  | [0.522] | [0.509] | [0.537] |
| BIC | -39294.246 | -39288.996 | -39298.791 |
| Log likelihood | -3080.747 | -3083.372 | -3061.3148 |

Source: WageIndicator data for Belgium, Germany and the Netherlands, 2006 – 2012 (unweighted), N=5,323

Note: Standard errors in brackets, * p < 0.05, ** p < 0.01, *** p < 0.00.
